# Delta-secretase triggers Alzheimer’s disease pathologies in wild-type hAPP/hMAPT double transgenic mice

**DOI:** 10.1038/s41419-020-03270-7

**Published:** 2020-12-12

**Authors:** Zhourui Wu, Xia Liu, Liming Cheng, Keqiang Ye

**Affiliations:** 1 Department of Pathology and Laboratory Medicine, Emory University School of Medicine, Atlanta, GA 30322 USA; 2 Division of Spine, Department of Orthopedics, Tongji Hospital affiliated to Tongji University School of Medicine, Shanghai, 200065 China; 3 Key Laboratory of spine and spinal cord injury repair and regeneration, Ministry of Education of the People’s Republic of China, Shanghai, 200072 China

**Keywords:** Alzheimer's disease, Neurological disorders

## Abstract

Alzheimer’s disease (AD) is the most common neurodegenerative disease with multifactorial pathologies including Aβ containing senile plaques and neurofibrillary tangles (NFT) consisted of aggregated Tau. Most of the AD patients are sporadic and the familial mutation hereditary patients are composed only 1% of all cases. However, the current AD mouse models employ mutated APP, PS1, or even Tau mutant, in order to display a portion of AD pathologies. Delta-secretase (legumain, or asparaginyl endopeptidase, AEP) simultaneously cleaves both APP and Tau and augments Aβ production and Tau hyperphosphorylation and aggregation, contributing to AD pathogenesis. Here we show that δ-secretase is sufficient to promote prominent AD pathologies in wild-type hAPP/hMAPT double transgenic mice. We crossed hAPP l5 mice and hMAPT mice to generate double transgenic mouse model carrying both human wild-type APP and Tau. Compared to the single transgenic parents, these double transgenic mice demonstrated AD-related pathologies in one-year-old hAPP/hMAPT mice. Notably, overexpression of δ-secretase in hAPP/hMAPT double-transgenic mice evidently accelerated enormous senile plaques and NFT, associated with prominent synaptic defects and cognitive deficits. Hence, δ-secretase facilitates AD pathogenesis independent of any patient-derived mutation.

## Introduction

Alzheimer’s disease (AD), an age-dependent neurodegenerative disorder with progressive cognitive impairments, is characterized by the accumulation of the extracellular β-amyloid peptide (Aβ) within the brain along with hyperphosphorylated and cleaved forms of the microtubule-associated protein, Tau, as the pathological hallmarks. It is known that metabolic dysfunction of amyloid-β precursor protein (APP) and abnormal intraneuronal Tau protein phosphorylation leads to the formation of senile plaques and neurofibrillary tangles (NFT), respectively. AD is a complex and heterogeneous disorder, and several factors play critical roles in its pathogenesis^1^. The defining pathologic features of amyloid plaques and NFTs are strongly intertwined with chronic neuroinflammation and extensive neuronal degenerative events^2^. Hippocampus, especially the CA1 region is responsible for learning and memory, which displays the main pathologies and responsible for dementia, the most critical symptoms in Alzheimer’s disease^3,4^. Despite considerable research efforts in the field, the pathogenesis of AD remains obscure. Although numerous theories including an amyloid cascade hypothesis have been proposed^5^, none of the propositions temporally and spatially elucidates the AD pathology initiation and progression^6,7^.

Mammalian asparaginyl endopeptidase (AEP, gene name: *LGMN*) is an endo-lysosomal cysteine protease that cleaves after asparagine residues. We show that AEP acts as δ-secretase that proteolytically cleaves both APP and Tau, and these proteins were fragmented by δ-secretase in human AD brains^8^. Delta-secretase autocleavage and activation is induced by pH reduction in the brain during aging. Interestingly, δ-secretase expression levels and activity are escalated in aged mice and human AD and PD brains^9,10^. Most recently, we report that C/EBPβ, an inflammatory cytokine-mediated transcription factor, regulates δ-secretase expression in an age-dependent manner in AD and PD brains^11,12^. δ-secretase cuts APP at both N373 and N585 residues in the ectodomain, and facilitates Aβ production by decreasing the steric hindrance for β-amyloid site cleaving enzyme 1 (BACE1). The depletion of δ-secretase significantly reduces Aβ production and senile plaque formation in 5XFAD mouse brains, leading to the restoration of synaptic activity and cognitive functions^10^. Additionally, δ-secretase cuts Tau at N255 and N368 residues and abolishes its microtubule assembly activity, resulting in its aggregation and NFT formation. Notably, the cleaved Tau 1-368 fragment is neurotoxic. The deletion of δ-secretase from Tau P301 mice reverses the synaptic plasticity defect and cognitive dysfunctions^9^. Co-expression of δ-secretase-truncated human APP C586-695 fragment and human Tau N368 truncate in the hippocampus of wild-type mice additively drives AD-like pathogenesis and cognitive dysfunctions^13^, suggesting that δ-secretase activity may promote AD-like pathology onset when both APP and Tau are abundant. Recently, we report that SRPK2, a cell-cycle-regulated kinase, phosphorylates S226 residue on δ-secretase and activates the enzyme, triggering its cytoplasmic translocation from the lysosomes^14^. On the contrary, BDNF-mediated Akt phosphorylates T322 residue on δ-secretase and inactivates it, sequestering its lysosomal residency^15^. Remarkably, Tau N368 fragments are demonstrable in the CSF and tissue lysate of human AD patients. Concentrations of Tau N368 in these CSF samples are both correlated tightly with Tau in PET images from AD brains and related to the extent of disease deterioration, indicating that δ-secretase is proteolytically activated in AD patient brains and mediates Tau pathologies^16^, fitting with the observations that abnormal Tau hyperphosphorylation and deposition are better related to disease severity and cognitive disorders^17^. Taken together, these findings support the hypothesis that δ-secretase plays a pivotal role in AD pathogenesis. In the present study, we established a new mouse AD model with both human wild-type APP and Tau (hAPP/hMAPT). Remarkably, overexpression of δ-secretase instigates the formation of senile plaques and NFTs in these mice and incurs an extensive neuronal loss and prominent neuro-inflammation, leading to demonstrable cognitive dysfunctions.

## Results

### Characterization of hAPP/hMAPT double-transgenic mice

**Fig. 1:**
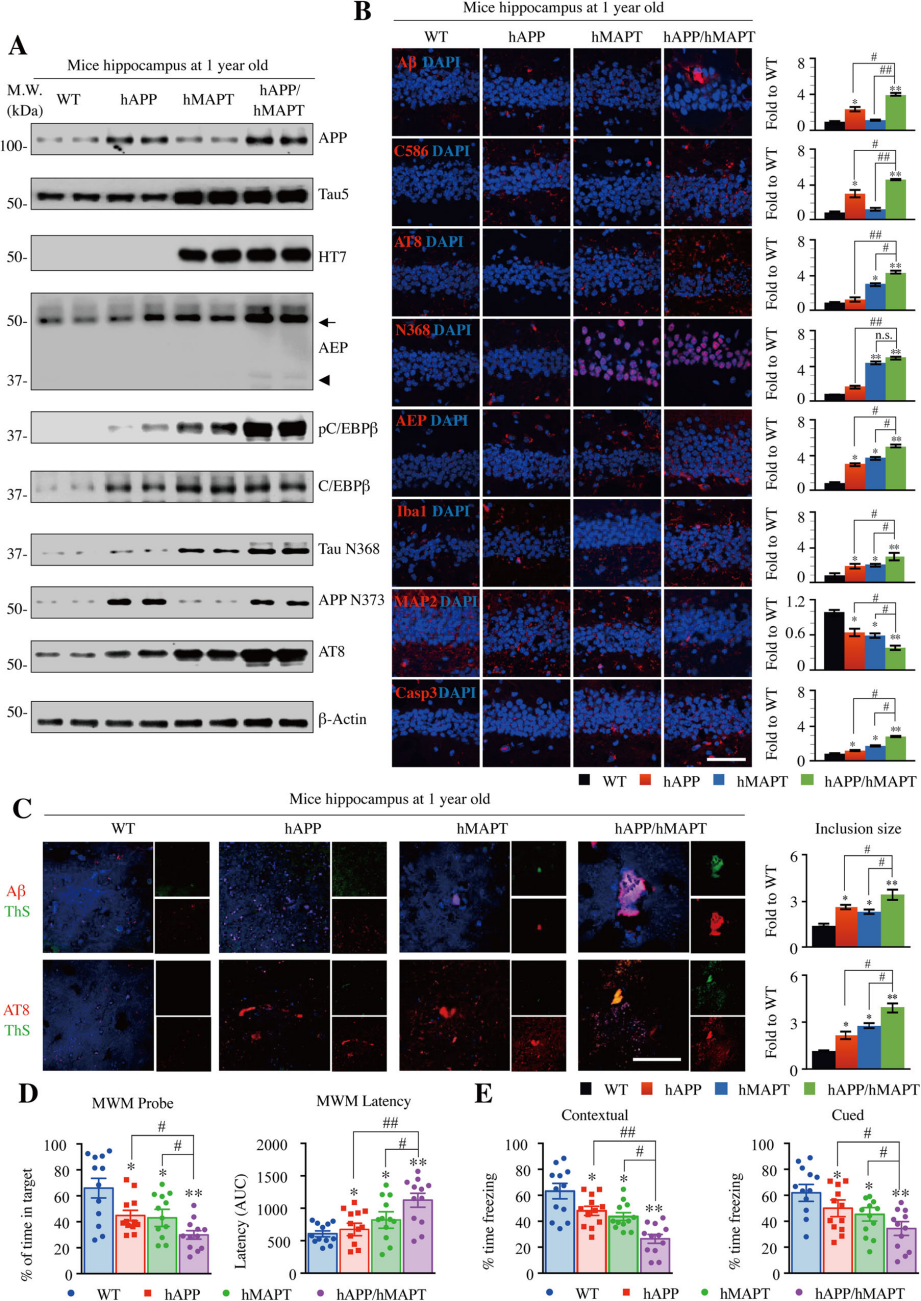
Characterization of hAPP/hMAPT double-transgenic mice. **A** Western blot assays demonstrated AD-related protein levels in human wild-type APP and human wild-type Tau double-transgenic mice, a new AD mouse model. Wild-type mice, human wild-type APP transgenic mice, human wild-type Tau single transgenic mice, human wild-type APP, and Tau double-transgenic mouse hippocampus were collected at 12 months of age. APP NT (for both mouse and human APP), Tau5 (for both mouse and human Tau), HT7 (for human Tau) antibodies were applied to validate the double-transgenic model. Arrow indicated full-length AEP (~56 kDa), arrowhead indicated activated (mature) AEP (~37 kDa). Quantitative analysis of relative mRNA levels can be found Supplementary Figure 1. **B** Immunofluorescent staining showed AD-like pathologies in the WT, two single transgenic lines, and human APP/MAPT mice, including amyloid deposition (Aβ antibody), Tau hyperphosphorylation (AT8 antibody), neuroinflammation (Iba1 antibody) and the dendritic reduction in the remained neurons (MAP2) as well as cleaved-Caspase3 (Casp3). The specific antibodies of AEP-cleaved APP (C586) and Tau (N368) were used for the detection of AEP activation (left, sale bar: 50 μm). Quantitative analysis the percentage of positive cells. The number of Iba1+ microglia cell body was count and then calculated as mean±SEM × 10^3^/mm^3^. (right, Mean±SEM, *n* = 6 mice for each group, one-way ANOVA with Dunnett’s multiple-comparisons test. * as compared to WT mice, # as comparison to hAPP/hMAPT mice). **C** The formation of amyloid plaques and neurofibrillary tangle (NFT) in four lines of mice at one-year old were shown by co-staining of ThS + Aβ and ThS + AT8, respectively (left. Scale bar: 50 μm). Quantitative analysis to demonstrate the size of Aβ + plaque and AT8 + NFT (right. Mean±SEM, *n* = 6 mice in each group, one-way ANOVA with Dunnett’s multiple-comparisons test, * as compared to WT mice, # as comparison to hAPP/hMAPT mice). **D** Morris water maze analysis as the percentage of time spent in the target quadrant in the probe trial (left) and the latency time (right, AUC: area under the curve) to the platform in the training days. (Mean ± SEM, *n* = 12 mice for each group, one-way ANOVA with Dunnett’s multiple-comparisons test, * as compared to WT, # as comparison to hAPP/hMAPT mice). **E** Fear conditioning tests. Contextual (left) and cued (right) fear conditioning were time-dependently impaired in mice after the virus. (Mean ± SEM, *n* = 12 mice for each group, one-way ANOVA with Dunnett’s multiple-comparisons test, * as compared to WT mice, # as comparison to hAPP/hMAPT mice).

Currently, all of AD mouse models employ various human AD patient-derived APP mutants, or even combined with Tau P301 mutants, in order to recapitulate the senile plaques and NFT pathologies. Recently, we report that co-expression of δ-secretase-cleaved human APP C586-695 and Tau 1-368 fragments in the hippocampus of wild-type mice elicits demonstrable senile plaques and NFT, associated with evident neuronal loss and neuroinflammation, leading to cognitive disorders^13^. This finding suggests that δ-secretase activity might be sufficient for initiating AD-like pathologies in the presence of abundant APP and Tau substrates. To test this possibility, we crossed the human wild-type APP transgenic I5 mice^18^ with human wild-type MAPT transgenic mice (htau)^19^ to obtain hAPP/hMAPT double-transgenic mice. Immunoblotting revealed that both human APP and human Tau are highly upregulated in double-transgenic mice, associated with C/EBPβ upregulation and δ-secretase escalation, as compared to WT control, hAPP or hMAPT single transgenic mice (Fig. 1A, 1^st^ - 6^th^ panels). The upregulated δ-secretase was proteolytically cleaved and active, accompanied by demonstrable APP N373 and Tau N368 in double-transgenic mice, which were barely detectable in other mice. These two truncates were also demonstrable in the corresponding single transgenic mice. Accordingly, Tau was highly phosphorylated as revealed by the prominent AT8 signal in hAPP/hMAPT mice (Fig. 1A, 7^th^ - 9^th^ panels). Immunofluorescent (IF) staining showed that APP C586 was augmented in double-transgenic mice, associated with palpable Aβ activities. Moreover, Tau N368 and AT8 activities were increased in double-transgenic mice. These events were consistent with elevated δ-secretase levels. Further, expression of Iba1, a marker for microglial activation, was increased; by contrast, substantial neuronal loss and a pro-apoptotic trend were detected in hAPP/hMAPT double-transgenic mice, which was validated with enhanced active Caspase-3 expression (Fig. 1B). MAP2 staining in the remained neurons demonstrated a significant decrease in hMAPT single-transgenic and hAPP/hMAPT double-transgenic mice. In alignment with these observations, Aβ and Thioflavin S (ThS) double staining (Aβ/ThS) showed that augmented Aβ was aggregated into ThS-positive fibrils in the cortex of one-year-old double-transgenic mice. On the other hand, AT8/ThS co-staining indicated that detectable p-Tau in both hAPP and hMAPT single transgenic mice but only NFT stained with both AT8 and ThS were found in the cortex of one-year-old hAPP/hMAPT double-transgenic mice (Fig. 1C). Quantitative RT-PCR (qRT-PCR) showed that mouse APP mRNA levels remained comparable among these mouse strains. Mouse MAPT levels in hMAPT mice and hAPP/hMAPT mice were approximately half of those in WT or hAPP mice because endogenous mouse MAPT gene was knocked out in hMAPT mice (Supplementary Figure 1). Cognitive dysfunction was observed as assessed by Morris water maze and fear conditioning cognitive-behavioral tests when compared to age-matched wild-type mice. The impairments were the worst in hAPP/hMAPT double-transgenic mice (Fig. 1D), consistent with the most prominent AD-like pathologies in these double-transgenic animals. Hence, hAPP/hMAPT double-transgenic mice possess some of the AD-related pathologies that are absent in the single transgenic mice.

### Delta-secretase triggers AD-like pathologies and cognitive impairments in hAPP/hMAPT double-transgenic mice

**Fig. 2:**
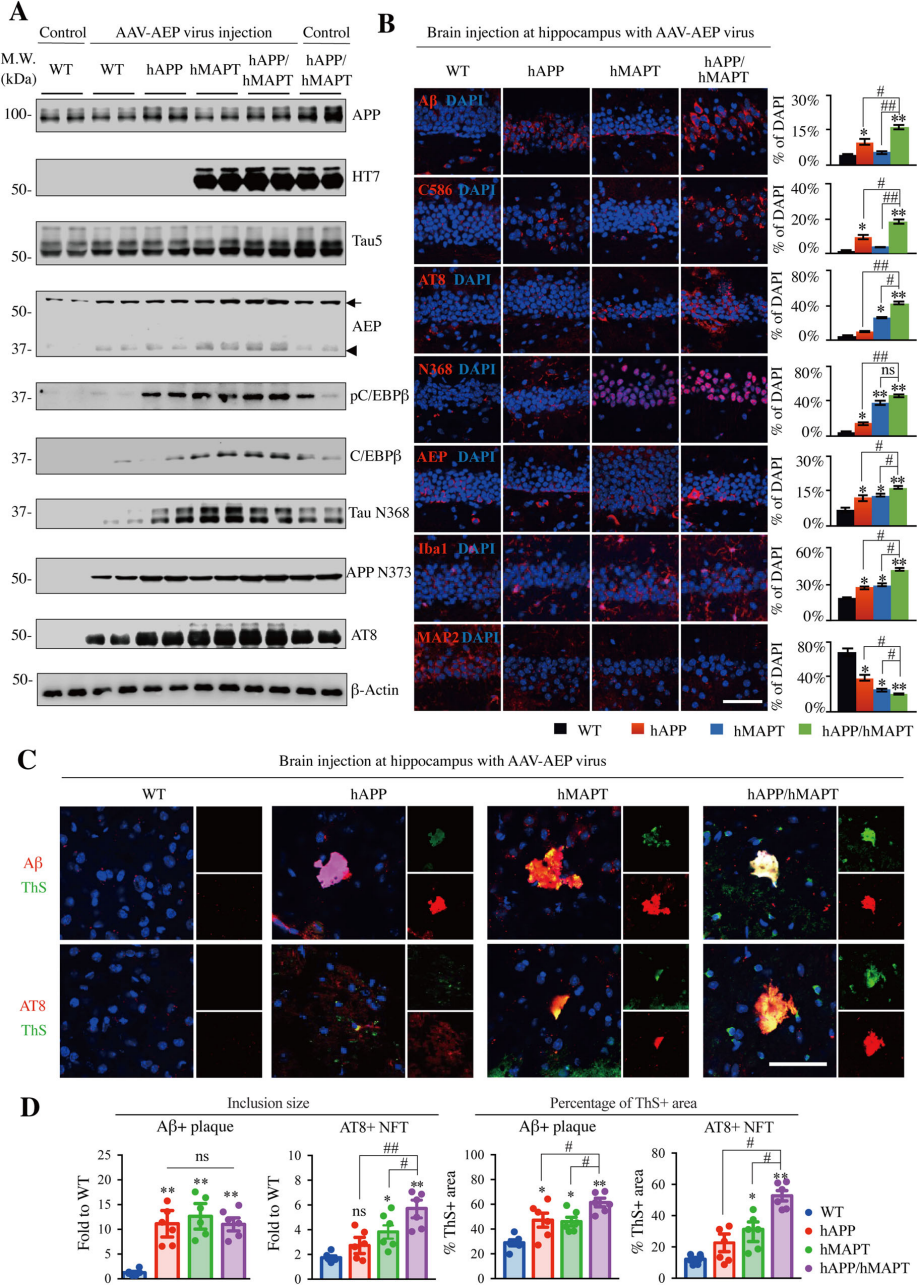
Delta-secretase enhances AD-like pathologies in hAPP/hMAPT double-transgenic mice. **A** δ-secretase overexpression induces APP and Tau proteolytic cleavage in the double-transgenic mice. Western blot assays demonstrated δ-secretase-cleaved APP, and Tau fragments promoted AD-like pathologies in human wild-type APP and Tau double-transgenic mice. Arrow indicated full-length AEP (~56 kDa), arrowhead indicated activated (mature) AEP (~37 kDa). WT, hAPP, hMAPT, hAPP/hMAPT mice at the age of 2-3 months old were injected with AAV-AEP virus in the bilateral hippocampus for 3 months. WT mice (lane 1 and 2) and hAPP/hMAPT mice (lane 11 and 12) with AAV-Control virus injection were compared. APP NT (for both mouse and human APP), Tau5 (for both mouse and human Tau), HT7 (for human Tau) antibodies were applied to validate the double-transgenic model. AEP (6E3) antibodies were used to verify the efficiency of AAV-AEP virus injection. **B** δ-secretase elicits AD-like pathologies in hAPP/hMAPT mice. Immunofluorescent staining showed AD-like pathologies in the WT, hAPP, hMAPT, and hAPP/MAPT mice injected with AAV-AEP virus, including amyloid deposition (Aβ antibody), Tau hyperphosphorylation (AT8 antibody), neuroinflammation (Iba1 antibody), and the dendritic reduction in the remained neurons (MAP2). The specific antibodies of AEP-cleaved APP (C586) and Tau (N368) were used for the detection of AEP activation (left, scale bar: 50 μm). Quantitative analysis the percentage of positive cells. The number of Iba1+ microglia cell bodies was count and then calculated as mean±SEM × 10^3^/mm^3^. (right, Mean±SEM, *n* = 6 mice for each group, one-way ANOVA with Dunnett’s multiple-comparisons test. * as compared to WT mice, # as comparison to hAPP/hMAPT mice). **C** The formation of amyloid plaques and neurofibrillary tangle (NFT) in four lines of mice treated with the AAV-AEP virus was shown by co-staining of ThS + Aβ and ThS + AT8, respectively. Scale bar: 50 μm. **D** Quantitative analysis to demonstrate the size of Aβ + plaque and AT8 + NFT (left, Mean±SEM, *n* = 6 in each group, one-way ANOVA with Dunnett’s multiple-comparisons test, * as compared to WT + AAV-AEP virus, # as comparison to hAPP/hMAPT + AAV-AEP virus) and the proportion (%) of Thioflavin S positive area (right). (Mean ± SEM, *n* = 6 in each group, one-way ANOVA with Dunnett’s multiple-comparisons test, * as compared to WT + AAV-AEP virus, # as comparison to hAPP/hMAPT + AAV-AEP virus).

To determine whether overexpression of δ-secretase is sufficient to trigger AD-like pathologies in hAPP/hMAPT double-transgenic mice, we injected the AAV9-AEP virus into the hippocampus of 2 month-old wild-types, hAPP, hMAPT and hAPP/hMAPT transgenic mice. Meanwhile, the AAV9-Control virus without overexpression of AEP was injected into the same location of 2 month-old wild-type and hAPP/hMAPT mice as the control for the effect of surgery and viral infection. There was no complication or abnormality after injection. No virus injection-caused AEP activation was observed in the AAV-Control group. In 3 months after injection, we monitored the expression of δ-secretase and various biochemical events by immunoblotting. Overexpressed δ-secretase was auto-cleaved and activated, which triggered APP cleavage into N373 and Tau into Tau N368 fragments, respectively, associated with robust Tau phosphorylation. Noticeably, expression of the inflammation- and Aβ-regulated transcription factor, C/EBPβ, was escalated, and it was also highly phosphorylated in δ-secretase-infected hAPP, hMAPT and the double-transgenic mice (Fig. 2A). IF analysis showed that overexpression of δ-secretase increased APP C586, Aβ, Tau N368, and AT8 signals, but these activities were all further augmented in hAPP/hMAPT double-transgenic mice, as were Iba1 immunoreactivity and neuronal loss. In hippocampus region, only intracellular Aβ rather than extracellular Aβ was found and then quantified (Fig. 2B). Surprisingly, extracellular insoluble Aβ-positive plaque was detected in the cortex region. As demonstrated in Fig. 2C, anti-Aβ/ThS and anti-AT8/ThS co-staining indicated that overexpression of δ-secretase increased double-positive signals in both hAPP and hMAPT single transgenic mice. The maximal effect occurred to hAPP/hMAPT double-transgenic mice, which exhibited Aβ aggregates that were ThS-positive, and AT8 inclusions that were also ThS-positive, respectively (Fig. 2C). Quantitative analysis for the inclusion sizes and the percentages of the ThS+ area in both Aβ + plaque and AT8 + NFT demonstrated that formations of amyloid plaque and NFT were accelerated in the double transgenic mice with overexpression of AEP (Fig. 2D). Thus, overexpression of δ-secretase strongly increases both APP and Tau cleavage, resulting in senile plaque deposition and NFT formation, which are associated with neuroinflammation and neuronal loss.

**Fig. 3:**
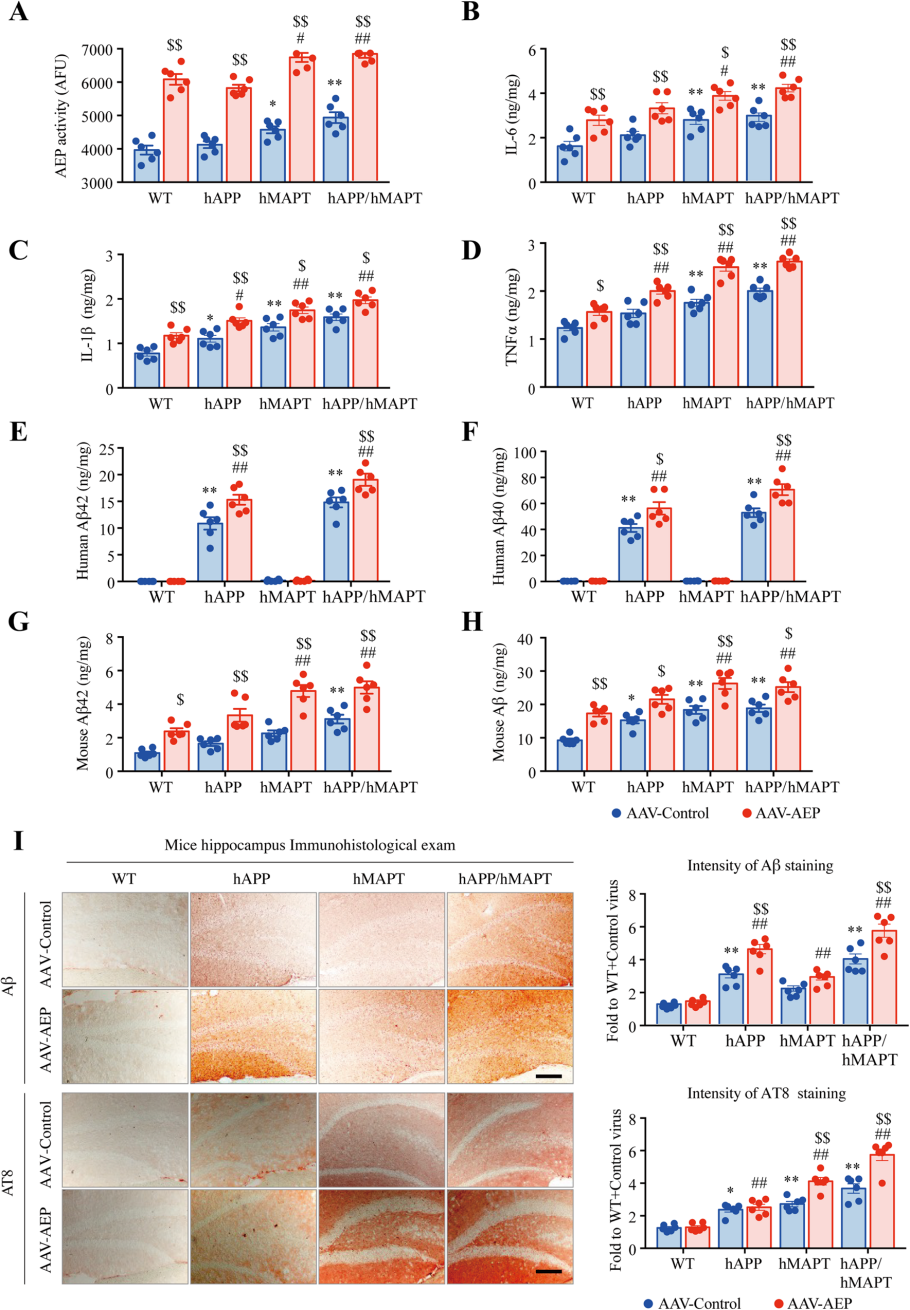
Overexpression δ-secretase in the hippocampus of hAPP/hMAPT mice induces Aβ escalation and neuroinflammation. **A** δ-secretase enzymatic assay. AEP enzymatic activity assay showed AEP activity in all the four lines of mice injected with AAV-AEP and AAV-Control virus. δ-secretase was increased in the hAPP/hMAPT mice, and overexpression of δ-secretase displayed the highest activities in hAPP/hMAPT double-transgenic mice (Mean ± SEM, *n* = 6 mice for each group, two-way ANOVA with Bonferroni multiple-comparisons test. * as compared to the WT mice + AAV-Control Virus; # as compared to the WT mice + AAV-AEP virus; $ as compared to AAV-Control virus in same mouse strain). **B** ELISA analysis of neuroinflammation factors IL-6 in the brain lysates from four lines of mice treated with AAV-AEP and AAV-Control virus (Mean ± SEM, *n* = 6 mice for each group, two-way ANOVA with Bonferroni multiple-comparisons test. * as compared to WT mice + AAV-Control virus; # as compared to the WT mice + AAV-AEP virus; $ as compared to AAV-Control virus in same mouse strain). **C** ELISA analysis of neuroinflammation factors IL-1β in the brain lysates from four lines of mice treated with AAV-AEP and AAV-Control virus (Mean ± SEM, *n* = 6 mice for each group, two-way ANOVA with Bonferroni multiple-comparisons test. * as compared to WT mice + AAV-Control virus; # as compared to the WT mice + AAV-AEP virus; $ as compared to AAV-Control virus in same mouse strain). **D** ELISA analysis of neuroinflammation factors TNFα in the brain lysates from four lines of mice treated with AAV-AEP and AAV-Control virus (Mean ± SEM, *n* = 6 mice for each group, two-way ANOVA with Bonferroni multiple-comparisons test. * as compared to WT mice + AAV-Control virus; # as compared to the WT mice + AAV-AEP virus; $ as compared to AAV-Control virus in same mouse strain). **E** ELISA analysis of human Aβ42 in the brain lysates from four lines of mice treated with AAV-AEP and AAV-Control virus. (Mean ± SEM, *n* = 6 mice for each group, two-way ANOVA with Bonferroni multiple-comparisons test. * as compared to WT mice + AAV-Control virus; # as compared to the WT mice + AAV-AEP virus; $ as compared to AAV-Control virus in same mouse strain). **F** ELISA analysis of human Aβ40 in the brain lysates from four lines of mice treated with AAV-AEP and AAV-Control virus. (Mean ± SEM, n = 6 mice for each group, two-way ANOVA with Bonferroni multiple-comparisons test. * as compared to WT mice + AAV-Control virus; # as compared to the WT mice + AAV-AEP virus; $ as compared to AAV-Control virus in same mouse strain). **G** ELISA analysis of mouse Aβ42 in the brain lysates from four lines of mice treated with AAV-AEP and AAV-Control virus. (Mean ± SEM, *n* = 6 mice for each group, two-way ANOVA with Bonferroni multiple-comparisons test. * as compared to WT mice + AAV-Control virus; # as compared to the WT mice + AAV-AEP virus; $ as compared to AAV-Control virus in same mouse strain). **H** ELISA analysis of mouse Aβ40 in the brain lysates from four lines of mice treated with AAV-AEP and AAV-Control virus. (Mean ± SEM, *n* = 6 mice for each group, two-way ANOVA with Bonferroni multiple-comparisons test. * as compared to WT mice + AAV-Control virus; # as compared to the WT mice + AAV-AEP virus; $ as compared to AAV-Control virus in same mouse strain). (**I**) Immunohistochemistry characterization of Aβ and AT8. The hippocampal sections of the indicated mice infected with AAV-AEP and AAV-Control virus were stained with anti-Aβ or AT8. Overexpression of δ-secretase increased Aβ and p-Tau signals in the double-transgenic mice (left, scale bar: 30 μm). Quantitative analysis of the immunohistochemical signals in each group (right, Mean±SEM, *n* = 6 mice for each group, two-way ANOVA with Bonferroni multiple-comparisons test. * as compared to WT mice + AAV-Control virus).

Fitting with immunoblotting findings, δ-secretase enzymatic activity was the highest in the double-transgenic mice, relative to the other three mouse strains. Injection of AAV-AEP further escalated its enzymatic activities (Fig. 3A). Quantitative analysis of inflammatory cytokines including IL-1β, IL-6, and TNFα disclosed the same pattern. All were more abundant in double-transgenic mice after δ-secretase injection (Fig. 3B-D). Aβ ELISA showed that the most copious human Aβ 1-40 and 1-42 were found in hAPP/hMAPT mice, followed by hAPP transgenic mice (Fig. 3E, F). Levels of mouse Aβ1-40 or 1-42 concentrations also were the highest in hAPP/hMAPT mice after AAV-AEP injection (Fig. 3G, H). The absolute values of human Aβ40 and Aβ42 in hAPP mice were half of the ones in human wild-type APP mice (hAPP*wt* I5) mice strain at a similar age (the molecular weights of human Aβ40 and Aβ42 were 4514 g/mol and 4330 g/mol, respectively), demonstrating its heterozygous genetic background of human APP genes^18^. Even though, mouse Aβ40 and Aβ42 levels of hAPP mice were comparable to WT and hMAPT, indicating the neurotoxicity of exogenous human Aβ associated with the elevated AEP resulted in the accumulation of intrinsic mouse Aβ. Immunohistochemical (IHC) staining with anti-Aβ validated these observations (Fig. 3I, upper panels). AT8 staining showed p-Tau activities were the strongest in double-transgenic mice, which were further elevated upon δ-secretase overexpression (Fig. 3I, lower panels).

**Fig. 4:**
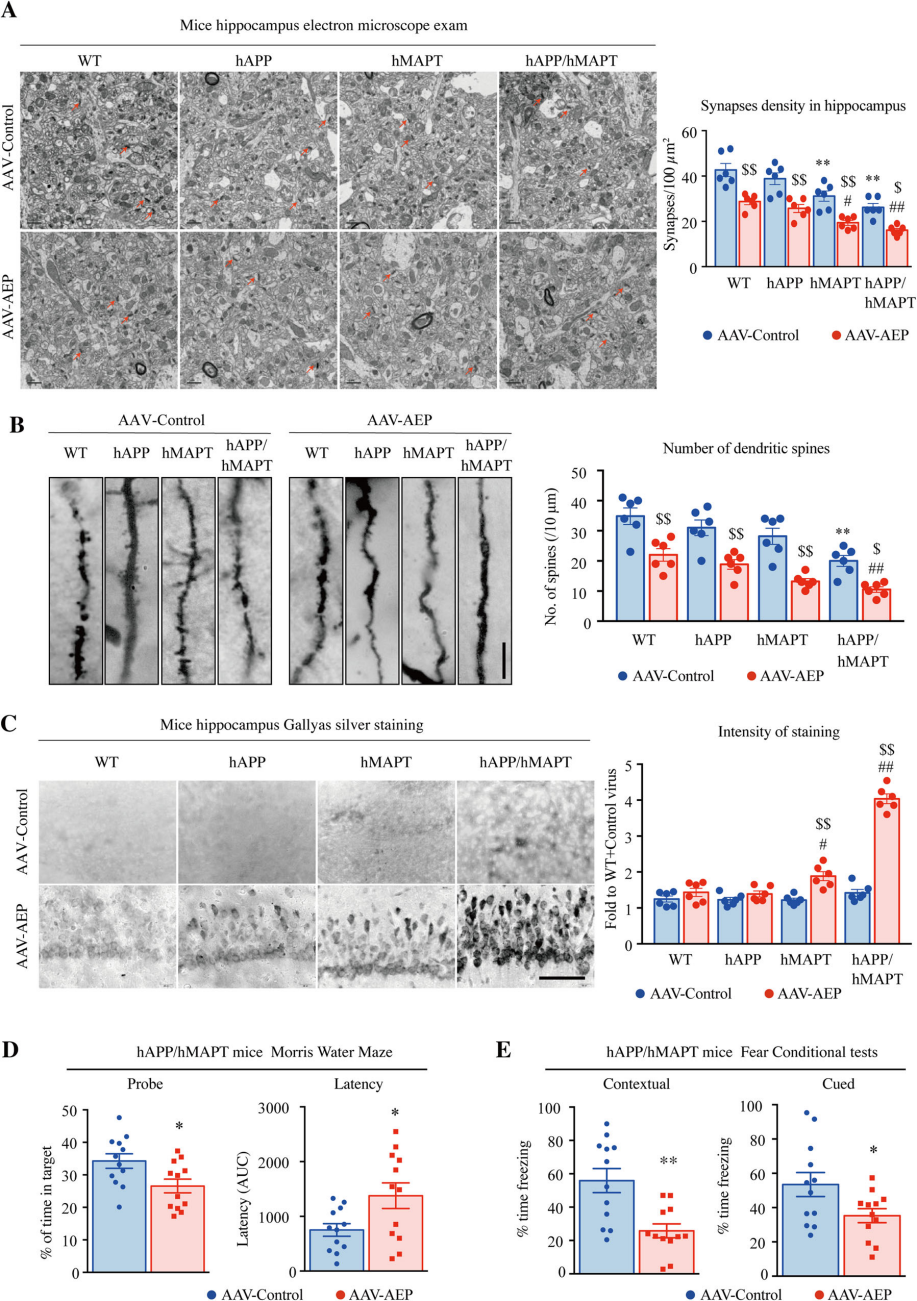
δ-secretase overexpression triggers cognitive dysfunctions, associated with synapse and dendritic spine loss in hAPP/hMAPT mice. **A** Representative electron microscopy images of the synaptic structures at the CA1 region at 3 months after the AAV-AEP virus injection. Arrows indicated the synapses (left, scale bar: 2 μm). Quantitative analysis of the synaptic density in each group (right, Mean±SEM, *n* = 6 mice for each group, two-way ANOVA with Bonferroni multiple-comparisons test. * as compared to WT mice + AAV-Control virus; # as compared to the WT mice + AAV-AEP virus; $ as compared to AAV-Control virus in same mouse strain). **B** Golgi staining showed the dendritic spines from the apical dendritic layer of the CA1 region. (Left, Scale bar: 5 μm). Quantitative analysis of the spine density. (Right, Mean±SEM, *n* = 6 mice for each group, two-way ANOVA with Bonferroni multiple-comparisons test. * as compared to WT mice + AAV-Control virus; # as compared to the WT mice + AAV-AEP virus; $ as compared to AAV-Control virus in same mouse strain). **C** Gallyas silver staining for senile plaque and NFT in the hippocampus (Left. Scale bar: 50 μm). Quantitative analysis of the percentage of signal intensity (right, Mean±SEM, *n* = 6 mice for each group, two-way ANOVA with Bonferroni multiple-comparisons test. * as compared to WT mice + AAV-Control virus; # as compared to the WT mice + AAV-AEP virus; $ as compared to AAV-Control virus in same mouse strain). **D** Morris water maze analysis as the percentage of time spent in the target quadrant in the probe trial (left) and the latency time (right, AUC: area under the curve) to the platform in the training days. (Mean ± SEM, *n* = 12 mice for each group, unpaired t-test, two-tailed, * as compared to hAPP/hMAPT mice + AAV-Control virus). **E** Fear conditioning tests. Contextual (left) and cued (right) fear conditioning were time-dependently impaired in mice after the virus injection. (Mean ± SEM, *n* = 12 mice for each group, unpaired t-test, two-tailed, * as compared to hAPP/hMAPT mice + AAV-Control virus).

Electron microscopic (EM) analysis indicated that apparent synapse loss was the greatest in double-transgenic mice, which was gradually reduced from hMAPT to hAPP to WT mice. Overexpression of δ-secretase stimulated the reduction of synapses. The fewest synapses were found in hAPP/hMAPT mice after δ-secretase overexpression (Fig. 4A). A similar pattern was observed in dendritic spines in these mice by Golgi staining (Fig. 4B). As above, overexpression of δ-secretase greatly reduced the dendritic spines in hAPP/hMAPT mice. In alignment with these observations, Gallyas silver staining revealed that the pathological aggregation increased in hAPP/hMAPT double-transgenic mice (Fig. 4C, upper panels), fitting with the findings by anti-Aβ/ThS and AT8/ThS co-staining in Fig. 2C. Overexpression of δ-secretase substantially enhanced this effect in both hAPP and hMAPT single transgenic mice with the climax effect that occurred to hAPP/hMAPT mice (Fig. 4C, lower panels). The behavioral tests showed that δ-secretase elicited significant cognitive impairments in hAPP/hMAPT mice compared to the control virus, consistent with the prominent AD-like pathologies in these mice (Fig. 4D, E). Collectively, these studies demonstrate that overexpression of δ-secretase elicits both APP and Tau cleavage and leads to AD-like pathologies and cognitive impairments in hAPP/hMAPT double-transgenic mice regardless of APP or Tau mutation.

## Methods

### Animals

Wild-type C57BL/6 J, hAPP (hAPPwt line I5), and hMAPT (htau) mice were purchased from the Jackson Laboratory (MMRRC stock # 000664, #004662, #005491, respectively). Human wild-type APP/MAPT double-transgenic mice (hAPP/hMAPT) were generated by crossing hAPP (hAPPwt line I5) and hMAPT (htau) mice. Human MAPT genes in both hMAPT and hAPP/hMAPT mice are heterozygous (±) since the homozygous insert is embryonic lethal^19^. Animal care and handling were performed according to the Declaration of Helsinki and Emory Medical School guidelines. Both male and female mice were used in the experiments. Mice were randomized into different groups by using a random number table. Investigators were blinded to the group allocation during the animal experiments.

### AAV vector packaging and stereotaxic injection

The AAV9 particles encoding AEP with CMV promoter were prepared by Viral Vector Core at Emory University. The AAV9 backbone plasmid without AEP cDNA was applied as the control for the effect of surgery and viral infection. Bilateral intracerebral injection of AAV-Control or AAV-AEP was performed stereotactically at coordinates posterior 1.94 mm, lateral 1.4 mm, ventral 2.2 mm relative to Bregma. 2 μl of viral suspension containing 2 × 10^11^ vector genome (vg) was injected into each point using 10 μl glass syringes with a fixed needle.

### Immunoblotting analysis

Mouse brain tissue samples were lysed in lysis buffer (50 mM Tris, pH 7.4, 40 mM NaCl, 1 mM EDTA, 0.5% Triton X-100, 1.5 mM Na_3_VO_4_, 50 mM NaF, 10 mM sodium pyrophosphate, 10 mM sodium-glycerophosphate, supplemented with protease inhibitors cocktail) on ice for 30 min. The lysates were centrifuged for 10 minutes at 15,000 rpm. Protein concentration from the supernatant was determined using a Coomassie Brilliant Blue protein assay kit (Bio-Rad). The same amount of the supernatant was boiled in SDS loading buffer. After SDS-PAGE, the samples were transferred to a nitrocellulose membrane. The membranes were blocked in 5% non-fat milk for 1 hour at room temperature and then incubated with primary antibody at 4 °C overnight. Primary antibodies to the following targets were used: APP NT (Millipore, # MAB348), HT7 (Invitrogen, # MN1000), Tau5 (Millipore, # MAB361), C/EBPβ C19 (Santa Cruz, # sc-150), p-C/EBPβ T188/T235 (CST, # 3084), AEP (11B7 as a gift from Dr. Colin Watts), AT8 (Thermo, #MN1020) and β-Actin (Sigma-Aldrich, # A1978). Both Tau N368 and APP C586 antibodies were generated in our lab and characterized in our previous publications^9,10^. Then the blots were incubated with IRDye 800CW-conjugated affinity-purified anti-mouse or anti-rabbit IgG secondary antibody (Rockland). Immunoreactive bands were visualized using the Odyssey Infrared Imaging System (Licor Biosciences, Lincoln, NE, USA). ImageJ software was applied to quantify the immunoblotting intensity.

### Immunostaining and Thioflavin S staining

The primary antibodies used were Aβ (Sigma, # A8354), AEP (11B7 as a gift from Dr. Colin Watts), Tau N368, APP C586, Iba1 (Sako, #NCNP24), Map2 (Sigma, # AB15452), AT8 (Thermo, #MN1020) and cleaved-Caspase3 (CST, #9664). Immunochemistry staining was performed with the immunohistochemistry staining kit (Invitrogen). Briefly, 4% paraformaldehyde (PFA)-fixed mice brain frozen slides were treated with 3% hydrogen peroxide at room temperature for 10 minutes. Manufacturer-supplied blocking buffer (Invitrogen) was used for each reaction. Then the sections were incubated with primary antibodies overnight at 4 °C. Biotin-conjugated secondary antibodies (Jackson ImmunoResearch), streptavidin-conjugated HRP (Invitrogen) were applied to enhance the signals. For immunofluorescence staining, brain sections were incubated with an Alexa Fluor 594 conjugated isotype-specific secondary antibody (Jackson ImmunoResearch, West Grove, PA) for 1 hour at room temperature. To quantify and compare Aβ in CA1 region, intracellular Aβ was measured and Aβ-positive cells were counted. For Thioflavin S (ThS) + Aβ/AT8 double staining, slides were rinsed in PBS after finishing the Aβ/AT8 first antibodies and the second antibody staining. Freshly dissolved 3.125 mg Thioflavin S in 100 ml 50% Ethanol. Incubated the Aβ/AT8 stained slides in the ThS solution at room temperature for 7 minutes in the dark. Then wash the slides in 1% Triton-X-100 and then PBS three times to exclude the signal of soluble plaque and debris. The fluorescence staining was visualized with an Olympus confocal microscope. Immunostaining images and their colocalization were quantified using Volocity 6.3 from Perkin Elmer and Fiji/ImageJ Coloc 2, respectively. The quantitative analysis methods of ThS staining were applied as previously reported^20^.

### AEP activity assay

Tissue homogenates or cell lysates (50 μg) were incubated in 100 μL reaction buffer (20 mM citric acid, 60 mM Na_2_HPO_4_, 1 mM EDTA, 0.1% CHAPS, and 1 mM DTT, pH 5.5) containing 10 μM AEP substrate Z-Ala-Ala-Asn-AMC (Bachem). AMC released by substrate cleavage was quantified by measuring at 460 nm in a fluorescence plate reader at 37 °C in kinetic mode.

### ELISA quantitative analysis of Aβ

To detect the concentration of Aβ in total brain lysates, the mouse brains were homogenized in 8× mass of 5 M guanidine HCl/50 mM Tris-HCl (pH 8.0) and incubated at room temperature for 3 h. Then the samples were diluted with cold reaction buffer (PBS with 5% BSA and 0.03% Tween-20, supplemented with protease inhibitor cocktail), and centrifuged at 16,000 g for 20 min at 4 °C. The Aβ in the total brain was analyzed with human Aβ42 (KHB3441, Invitrogen), human Aβ40 (KHB3481, Invitrogen), mouse Aβ42 (KMB3441, Invitrogen), and mouse Aβ40 (KMB3481, Invitrogen) ELISA kits according to the manufacturer’s instructions. The Aβ concentrations were determined by comparison with the standard curve.

### Real-time PCR

Levels of mRNA were analyzed by real-time, quantitative PCR. RNA was isolated by Trizol (Life Technologies). Reverse transcription was performed with SuperScriptIII reverse transcriptase (Life Technologies). Gene-specific primers and probes were designed and bought from Taqman (Life Technologies). All real-time PCR reactions were performed using the ABI 7500-Fast Real-Time PCR System and TaqMan Universal Master Mix Kit (Life Technologies). The relative quantitative analysis of gene expression was calculated as the 2-▵▵Ct method. For each data point, at least duplicated wells were used. GAPDH was used for housekeeping gene control. And each experiment was repeated at least 3 times.

### Golgi staining

Mouse brains were fixed in 10% formalin for 24 h and then immersed in 3% potassium dichromate for 3 days in the dark. The solution was changed each day. Then the brains were transferred into 2% silver nitrate solution and incubated for 24 h in the dark. Vibratome sections were cut at 60 μm, air-dried for 10 min, dehydrated through 95 and 100% ethanol, cleared in xylene and cover-slipped. Bright-field images of pyramidal neurons in the hippocampus and cortex were taken at 100× magnification using a Zeiss Axioplan (Zeiss, Decatur, GA, USA) microscope. To measure the spine density, all clearly evaluable areas of 50–100 μm of the secondary dendrites from each imaged neuron were used.

### Electron microscopy

Synaptic density was determined by electron microscopy. After deep anesthesia, mice were perfused transcardially with 2% glutaraldehyde and 3% paraformaldehyde in PBS. Hippocampal slices were post-fixed in cold 1% OsO4 for 1 h. Samples were prepared and examined using standard procedures. Ultrathin sections (90 nm) were stained with uranyl acetate and lead acetate and viewed at 100 kV in a JEOL 200CX electron microscope. Synapses were identified by the presence of synaptic vesicles and postsynaptic densities.

### Morris Water Maze

Mice were trained in a round, water-filled tub (52-inch diameter) in an environment rich with extra-maze cues. An invisible escape platform was located in a fixed spatial location 1 cm below the water surface independent of subjects start position on a particular trial. In this manner, the subjects needed to utilize extra-maze cues to determine the platform’s location. At the beginning of each trial, the mouse was placed in the water maze with their paws touching the wall from 1 of 4 different starting positions (N, S, E, W). Each subject was given 4 trials/day for 5 consecutive days with a 15-min inter-trial interval. The maximum trial length was 60 s, and if subjects did not reach the platform in the allotted time, they were manually guided to it. Upon reaching the invisible escape platform, subjects were left on it for an additional 5 s to allow for the survey of the spatial cues in the environment to guide future navigation to the platform. After each trial, subjects were dried and kept in a dry plastic holding cage filled with paper towels to allow the subjects to dry off. The temperature of the water was monitored every hour so that mice were tested in water that was between 22 and 25 °C. Following the 5 days of task acquisition, a probe trial was presented during which time the platform was removed and the percentage of time spent in the quadrant which previously contained the escape platform during task acquisition was measured over 60 s. All latency trials were analyzed with MazeScan (Clever Sys, Inc.).

### Fear conditional tests

The ability to form and retain an association between an aversive experience and environmental cues was tested with a standard fear conditioning paradigm that occurs for 3 days. Mice were placed in the fear conditioning apparatus (7″ W, 7″ D 3 12″ H, Coulbourn) composed of Plexiglass with a metal shock grid floor and allowed to explore the enclosure for 3 min. Following this habituation period, 3 conditioned stimuli (CS)-unconditioned stimulus (US) pairings were presented with a 1 min intertrial interval. The CS was composed of a 20 s, 85-dB tone, and the US was composed of 2 s of a 0.5-mA footshock, which was co-terminate with each CS presentation. One minute following the last CS-US presentation, mice were returned to their home cage. On day 2, the mice were presented with a context test, during which subjects were placed in the same chamber used during conditioning on day 1, and the amount of freezing was recorded via a camera and the software provided by Coulbourn. No shocks were given during the context test. On day 3, a tone test was presented, during which time subjects were exposed to the CS in a novel compartment. Initially, animals were allowed to explore the novel context for 2 min. Then the 85-db tone was presented for 6 min, and the amount of freezing behavior was recorded.

### Experimental design and statistical analysis

All experiments were designed as described previously^21^ to provide sufficient power (80–90%) to discriminate significant differences (α = 0.05) in mean±standard error of the mean (SEM) between independent controls and experimental subjects. The numbers of tested subjects (n) were indicated in each figure legends. Statistical evaluations were performed with Student’s t-test, one-way or two-way ANOVA, as indicated in figure legends. Post-ANOVA comparisons were made using the Dunnett’s (one-way ANOVA) and Bonferroni (two-way ANOVA) correction. *p* values < 0.05 (indicated as *, #, $) and < 0.01 (indicated as **, ##, $$) were considered as statistically significant, ns indicates not significant.

## Discussion

In the current study, we show that viral expression of δ-secretase elicits AD-like pathologies and cognitive disorders in hAPP/hMAPT double-transgenic mice. It is worth noting that hAPP/hMAPT mice possess no mutation in the human APP or MAPT gene. Strikingly, overexpressed δ-secretase potently enhances Aβ production and senile plaques deposits, accompanied by robust APP N373 fragmentation in both hAPP and hAPP/hMAPT transgenic mice, though APP N373 appeared less abundant in double transgenic line compared to hAPP mice (Fig. 1A), which might be due to its further proteolytically cleavage into even smaller fragment by other strongly activated proteases. Moreover, it also leads to the formation of prominent NFT in hMAPT and hAPP/hMAPT mice, accompanied by potent Tau N368 truncation. Accordingly, hippocampal neuronal cell death was manifested in δ-secretase-injected hAPP/hMAPT double-transgenic mice. On the other hand, among the tested strains of mice, neuro-inflammation upon δ-secretase expression was the greatest in hAPP/hMAPT double-transgenic mice, as was the cognitive dysfunction. In agreement with these observations, EM and Golgi staining demonstrated that synaptic loss and dendritic spine reduction were the maximal in δ-secretase-injected hAPP/hMAPT mice compared with the other strains. Collectively, these findings strongly support the notion that δ-secretase is sufficient for initiating AD-like pathogenesis when the substrates of APP and Tau are abundant. Remarkably, it triggers both senile plaques and NFT pathologies independent of any AD patient-derived mutation. This finding provides an innovative insight into the molecular mechanisms in sporadic AD pathogenesis.

Since the etiology of AD is not completely known, a wide range of animal models has been developed to study the pathological processes based on APP and Tau which are two major pathological proteins found in AD onset^22^. APP is not only important for CNS maturation but also plays a role in cell contact and adhesion, in neuronal morphogenesis, in the maintenance of synaptic transmission and plasticity^23,24^. Accordingly, APP knockout mice develop behavioral and cognitive impairment^25,26^. APP overexpression results in an increased generation of toxic derivatives, Aβ peptide, and/or CTFs (C-terminal fragments). Moreover, APP overproduction, either as a result of genomic locus duplication in Down Syndrome or altered regulatory sequences in the APP promoter region, leads to early-onset AD in humans^27,28^. To explore the pathological roles of human APP in AD, several transgenic mice with wild-type human APP gene have been developed. For instance, Mucke and his colleagues reported that human wild-type APP overexpression mice (hAPP_wt_ I5) do not develop plaques, but show significant decreases in SYN-IR (synaptophysin-immunoreactivity) presynaptic terminals that worsen with age. Thus, FAD (familial AD) mutations are not required for the decrease in SYN-IR presynaptic terminals in hAPP mice, consistent with the loss of these structures in sporadic AD patients, who also lack FAD mutations. Loss of SYN-IR presynaptic terminals and the number of NFT in specific brain regions correlate well with cognitive decline in AD^29,30^. Moreover, Frenchilla et al. also report that hAPP transgenic mice display early memory deficits in mice overexpressing wild-type human APP despite almost undetectable Aβ42 levels in the hippocampus. However, an early increase in the levels of p-Tau in the hippocampus is manifested. In these mice, hAPP processing is non-amyloidogenic, with high levels of APP CTFs, C83, and APP intracellular domain (AICD). Furthermore, these mice present a loss of synapse-associated proteins. Importantly, signs of neurodegeneration are found in the hippocampal CA1 subfield and in the entorhinal cortex that is associated with a marked loss of MAP2 immunoreactivity, consistent with our observations described in Figs. 1 and 2. Not only is the number of neurons declined, but also the dendritic staining patterns are impaired. Immunostaining of MAP2 shows the number of branches negatively correlates with the AEP activation. This phenomenon is consistent with the results of Golgi staining which detects the dendritic spines. Conversely, in mice expressing mutant hAPP, high levels of Aβ42 are found in the hippocampus, but no signs of neurodegeneration are apparent^31^. The results support the notion of Aβ-independent pathogenic pathways in AD. It has been proposed that Aβ is synaptotoxic even in the absence of plaques and that high levels of Aβ42 are insufficient to induce plaque formation in mice expressing wild-type hAPP^18^. However, when we injected δ-secretase into the hippocampus of hAPP or hAPP/hMAPT transgenic mice, we observed not only human and mouse Aβ escalation (Fig. 3) but also senile plaques deposition in both mouse strains (Fig. 2), suggesting the overproduction of Aβ is prone to plaque formation and deposition.

Abnormal accumulation of wild-type human Tau proteins is a hallmark of sporadic AD^32,33^. Expression of full-length human Tau alone causes intracellular Tau pathologies and behavioral deficits in mice^19,34^ while turning off Tau expression attenuates the pathologies^35^. hMAPT (htau) mice undergo an age-related accumulation of AD-relevant phosphorylation on Tau in the cell bodies and dendrites of neurons and aggregated paired helical filaments (PHF) without exogenous factors^19^. Accumulation of hyperphosphorylated Tau begins by 6 months but increases further by 13 and 15 months of age. Aggregated Tau and PHFs are detectable in htau mice aged 9 months via the sarkosyl-insolubility assay and immunoelectron microscopy^19^. It is worth noting that some of Tau N368 resided in the nuclei (Fig. 2B). Nuclear tauopathy is believed to be highly correlated to neuronal degeneration^36^. Our Tau N368 antibody recognized the Tau N368 fragment generated by AEP cleavage, indicating the degeneration of CA1 neurons as well. We made similar observations in our previous report^9^. It should be pointed out that hMAPT mice lack endogenous mouse Tau. It has been proposed before that 4 R mouse Tau protects htau overexpression in wild-type mice (8c mice) from developing NFT pathology that results in hMAPT from the excess of human 3 R Tau. Nevertheless, in our hAPP/hMAPT double-transgenic mice, they also contain mouse Tau as well, like 8c mice. It is worth noting that htau mice do express the first 32 amino acids of the endogenous mouse tau; however, human tau-driven tauopathy overwhelmed the contribution of the endogenous mouse tau. We observed robust NFT pathology in 5 month-old hMAPT and hAPP/hMAPT mice, when δ-secretase is overexpressed. By contrast, these phenomena are alleviated in hAPP/hMAPT mice without overexpressing δ-secretase (Fig. 1). Therefore, these findings strongly support that δ-secretase is required for initiating AD-like pathologies, even though both wild-type human APP and human Tau are amply upregulated, highlighting that δ-secretase plays a key role in the initiation of AD pathogenesis.

## Conclusion

Currently, all AD mouse models are derived from APP, PS1, or Tau with patient-derived mutation, though familial AD cases are only 1% of total AD patients, and most AD patients are sporadic without any mutation. To address this need, we generated a human wild-type APP/MAPT double transgenic (hAPP/hMAPT) mouse model. Previously we have reported that δ-secretase cleaves APP and Tau and escalating Aβ and Neurofibrillary tangle (NFT) pathologies in AD pathologies. In this study, injection of δ-secretase into hAPP/hMAPT double transgenic mice elicits AD pathologies in the absence of any human patient-derived mutations, supporting that δ-secretase is sufficient to accelerate AD pathogenesis if both human wild-type APP and Tau substrates are sufficient. Thus, this innovative study demonstrates that δ-secretase plays a key role in driving AD onset.

## Supplementary information

Supplementary Figure Legends

Supplementary Figure 1

Supplementary Figure 2

Supplementary Figure 3

## Data Availability

The datasets used and/or analyzed during the current study are available from the corresponding authors on reasonable request.
