# Supplementary material for: Delta-secretase triggers Alzheimer’s disease pathologies in wild-type hAPP/hMAPT double transgenic mice
Source: Cell Death Dis. 2020 Dec 12;11(12):1058. doi: 10.1038/s41419-020-03270-7 (PMC7733592; doi:10.1038/s41419-020-03270-7)
Supplement: Supplementary file 1 — Supplementary Figure Legends [file 41419_2020_3270_MOESM1_ESM.docx]

**Supplementary Figure 1. Quantitative RT-PCR analysis of variance indicated genes in these transgenic mice**. The mRNA samples were extracted from the brains of WT, hAPP, hMAPT single transgenic mice and hAPP/hMAPT double-transgenic mice and analyzed with qRT-PCR. (Mean ± SEM, n = 4 mice in each group, one-way ANOVA with Dunnett’s multiple-comparisons test, * as compared to WT mice, # as comparison to hAPP/hMAPT mice).

**Supplementary Figure 2. Quantitative analysis of immunoblotting analysis supporting Figure 1A.** The immunoblotting intensity of each panel was measured with ImageJ software, followed with a normalization to its own β-Actin intensity. One-way ANOVA with Dunnett’s multiple-comparisons test, * as compared to WT mice.

**Supplementary Figure 3. Quantitative analysis of immunoblotting analysis supporting Figure 2A.** The immunoblotting intensity of each panel was measured with ImageJ software, followed with a normalization to its own β-Actin intensity. One-way ANOVA with Dunnett’s multiple-comparisons test, * as compared to hAPP/hMAPT mice + AAV-AEP virus.
