# Supplementary figures and images for: Delta-secretase triggers Alzheimer’s disease pathologies in wild-type hAPP/hMAPT double transgenic mice
Source: Cell Death Dis. 2020 Dec 12;11(12):1058. doi: 10.1038/s41419-020-03270-7 (PMC7733592; doi:10.1038/s41419-020-03270-7)

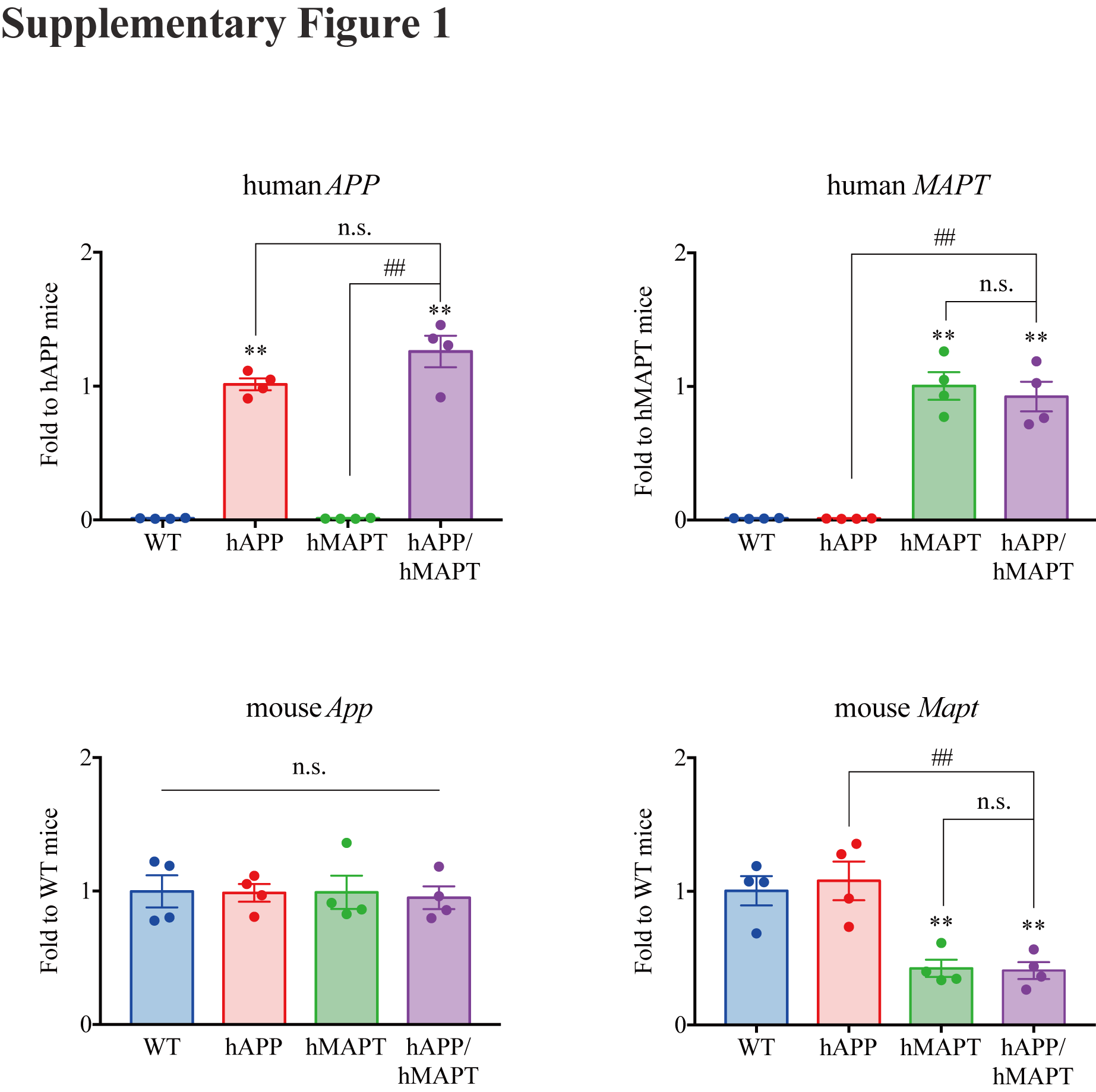

Supplement: Supplementary file 2 — Supplementary Figure 1 [file 41419_2020_3270_MOESM2_ESM.png]

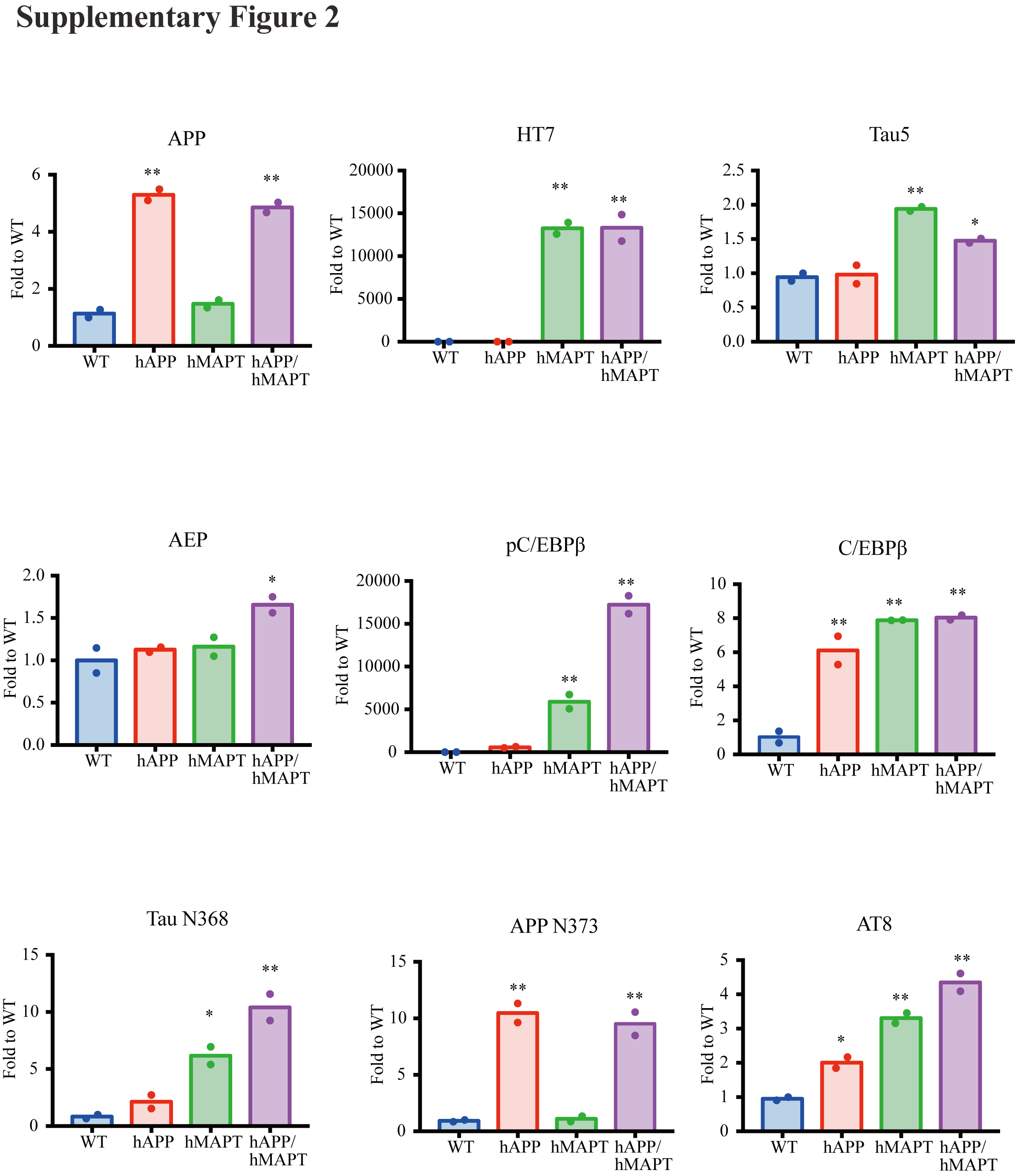

Supplement: Supplementary file 3 — Supplementary Figure 2 [file 41419_2020_3270_MOESM3_ESM.png]

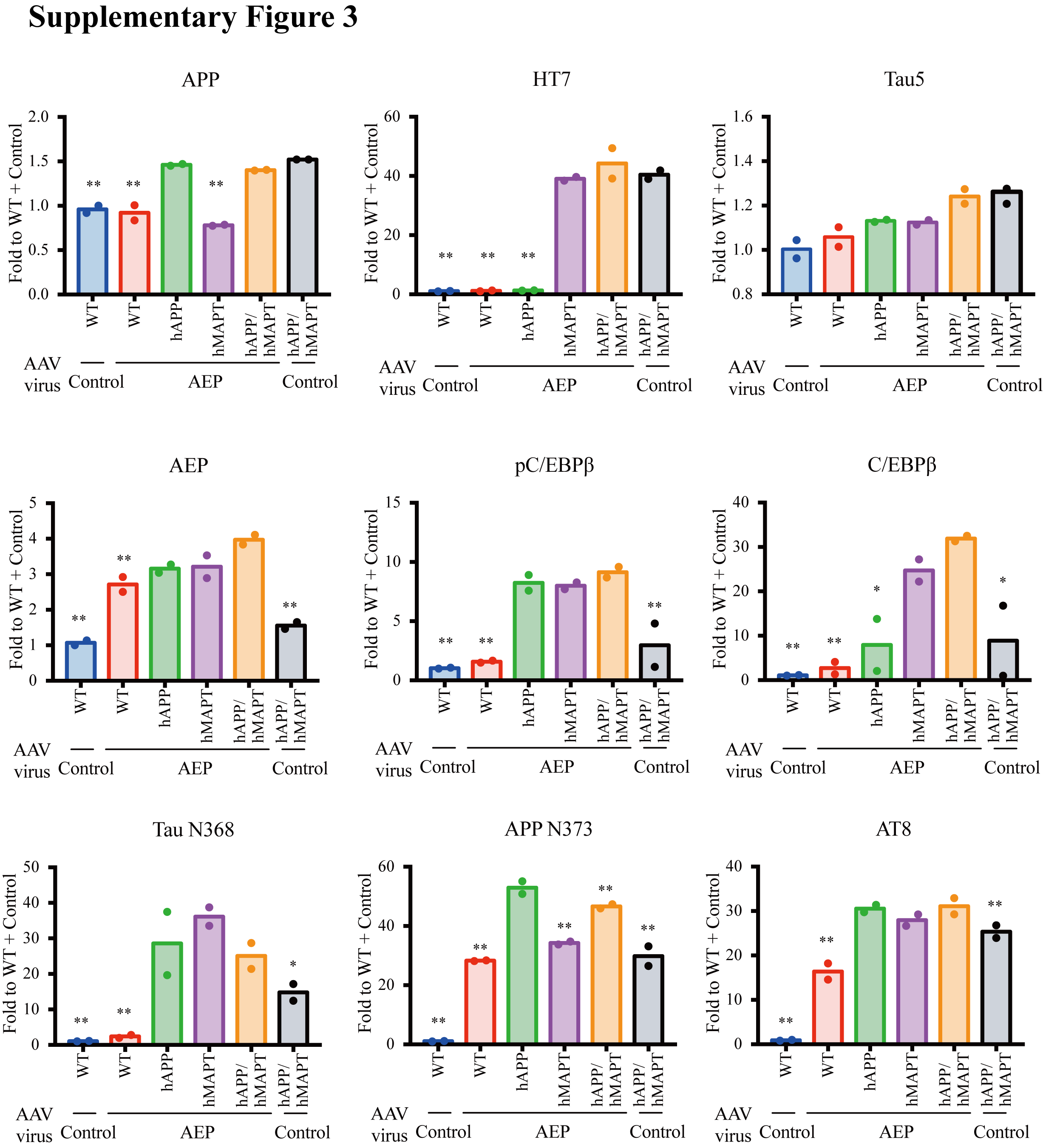

Supplement: Supplementary file 4 — Supplementary Figure 3 [file 41419_2020_3270_MOESM4_ESM.png]
